# Advancing Alzheimer’s Research With Zebrafish Models: Current Insights, Addressing Challenges, and Charting Future Courses

**DOI:** 10.7759/cureus.66935

**Published:** 2024-08-15

**Authors:** Shreya Dey, Tamilanban Thamaraikani, Chitra Vellapandian

**Affiliations:** 1 Pharmacy/Pharmacology, SRM College of Pharmacy, SRM Institute of Science and Technology, Chengalpattu, IND

**Keywords:** neuroinflammation, behavioral assays, pathological features, neurodegenerative disorders, crispr/cas9, genetic engineering, high-throughput screening, amyloid beta plaques, zebrafish models, alzheimer's disease (ad)

## Abstract

Alzheimer’s disease (AD) is a neurological condition that progressively impairs cognitive function and results in memory loss. Despite substantial research efforts, little is known about the specific processes driving AD, and there are few proven therapies. Because of their physiological and genetic resemblance to humans, zebrafish (*Danio rerio*) have become an important model organism for furthering research on AD. This abstract discusses the difficulties faced, looks at the insights currently garnered from zebrafish models, and suggests future research options. AD knowledge has greatly benefited from the use of zebrafish models. Transgenic zebrafish that express human AD-associated genes, such as tau and amyloid precursor protein (APP), display tau neurofibrillary tangles (NFTs) and amyloid-beta (Aβ) plaques, two of the disease’s main clinical characteristics. These models have clarified the roles of oxidative stress, inflammation, and calcium homeostasis in the course of AD and allowed for the purpose of high-throughput screening of potential therapeutic agents. Understanding the growth and deterioration of neurons has been greatly aided by real-time zebrafish imaging. Fully using zebrafish models in AD research requires addressing a number of issues. The dissimilarities in zebrafish anatomy and physiology from humans, the difficulty of developing models that replicate progressive and late-onset AD (LOAD), and the requirement for standardized procedures to evaluate alterations in zebrafish cognition and behavior are a few issues. Furthermore, variations in the genetic makeup of zebrafish strains might affect the results of experiments. Future directions include developing standardized behavioral assays and cognitive tests, working together to create extensive databases of zebrafish genetic and phenotypic data, and using genetic engineering techniques like CRISPR/Cas9 to create more complex zebrafish models. Combining zebrafish models with other model species helps expedite the conversion of research results into therapeutic applications and offers a more thorough knowledge of AD. To sum up, zebrafish models have made a substantial contribution to Alzheimer’s research by offering insightful information on the causes of the illness and possible therapies. By tackling present issues and formulating a planned future path, we can improve the use of zebrafish to decipher the mysteries of Alzheimer’s and help create successful treatments.

## Introduction and background

The world population is aging, and this is increasing the risk of neurodegenerative diseases (NDs), including amyotrophic lateral sclerosis (ALS), Parkinson’s disease (PD), and Alzheimer’s disease (AD), which are grave threats to human health. In addition to having serious social and economic repercussions, many age-related illnesses have a catastrophic impact on the individuals who are afflicted and their families. It is, therefore, imperative that new, more potent treatments for these conditions be developed. Since AD is the most common kind of dementia, an estimated 47 million individuals worldwide suffer from it. There will likely be a significant increase in the frequency of AD; 71 million cases are anticipated in the Asia-Pacific area alone. In all, 82 million people worldwide may have AD by 2030, and by 2050, that number might increase to 152 million. With present expenditures on AD estimated at $183 billion and projected to increase to $1.1 trillion by 2050, the economic burden of the disease is equally staggering [1].

**Figure 1:**
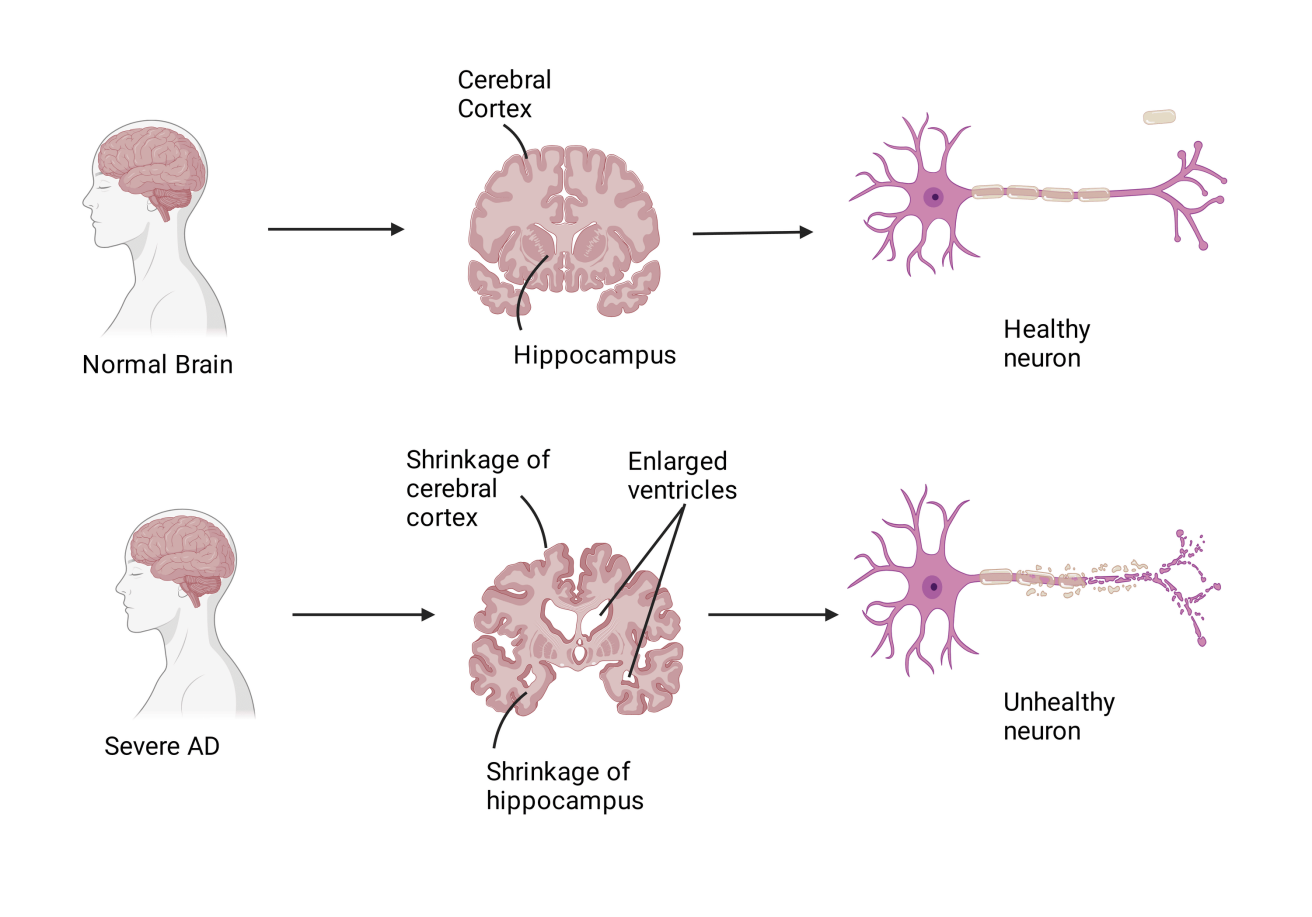
Difference between a healthy brain and Alzheimer’s disease brain This image was originally produced by the author. Created with Biorender.com.

The key features of AD include a significant loss of neurons and altered synaptic processes in the cerebral cortex (Figure 1), particularly in the frontal and temporal lobes and the hippocampus. AD is characterized by two main pathogenic features: internal hyperphosphorylated tau protein aggregates that form paired helical filaments that make up neurofibrillary tangles (NFTs) and the formation of extracellular amyloid-β (Aβ) peptide aggregates known as senile plaques. Numerous clinical symptoms are brought on by these pathological alterations, such as mood swings, difficulties speaking, eating, and walking, as well as cognitive impairment, memory loss, and disorientation. Even with much study, AD is still difficult to diagnose in its early stages, and significant neuronal loss in areas of the brain affected by the illness results in a discernible reduction in total brain capacity.

AD is classified into two primary types: sporadic AD (SAD) and familial AD (FAD). FAD is a hereditary type of illness that is often brought on by genetic mutations, whereas SAD arises sporadically and is not related to family history. The most common kind of AD has no clear inherited cause and often appears after age 65. It is the result of the complex interplay between genetic, lifestyle, and environmental factors. The APOE-4 ε4 allele is one genetic risk factor that may increase susceptibility; however, the inheritance pattern of SAD is unknown. FAD, an unusual genetically inherited type of AD, usually causes early-onset AD (EOAD) that appears before the age of 65. It is linked to mutations in genes such as APP, PSEN1, and PSEN2. Due to the autosomal dominant pattern of inheritance for FAD, inheriting a single mutant gene from either parent significantly increases the likelihood of developing the condition. The growing impact of AD on people, families, and society is a result of both kinds, which emphasizes the need for improvements in diagnostic and therapeutic methods. Even with a great deal of research, AD is still very difficult to treat. Although they have not yet resulted in treatment, the clinical indications of AD, such as Aβ plaques and NFTs in the brain, have been essential to understanding the illness’s progression, even if they haven’t led to a cure yet. The available therapies only partially relieve symptoms; they do not stop the underlying neurodegenerative processes. Therefore, in order to find promising medication candidates and get a deeper understanding of the illness processes, new therapeutic approaches and more effective screening techniques are crucial [2].

Zebrafish are a model organism that can be used to investigate AD, which is a promising line of inquiry. Because of their genetic resemblance to humans and their transparent embryos, which make it simple to see developmental processes, zebrafish are becoming more and more valued for their use in scientific research. Zebrafish have several benefits when it comes to AD research. They are a great platform for researching the illness since they have a similar neural network to that of humans. Zebrafish are a helpful resource for scholars to look at a range of AD-related characteristics, such as anxiety, cognitive decline, problems with spatial memory, and other behavioral alterations. Furthermore, the comparatively brief lifespan and rapid growth of zebrafish allow for the investigation of illness progression and the assessment of therapeutic treatments within a reduced timeframe. It is possible to analyze the development of NFTs and Aβ plaque formation in a live creature by genetically modifying zebrafish models to express human AD-related genes. Compared to traditional mammalian models, this makes it possible for researchers to more effectively and economically study the underlying molecular processes of AD and evaluate the effectiveness of possible medications.

Zebrafish models have the possibility for high-throughput chemical screening in addition to their practical benefits. This implies that it will be possible to swiftly screen through enormous libraries of putative treatment drugs to find those that have promise in modifying AD pathogenesis. By employing zebrafish in this manner, scientists can find novel medications more quickly and learn more about the basic mechanisms driving AD. Research on AD has advanced significantly with the use of zebrafish. In addition to offering insightful information about the mechanisms underlying disease, these models also tackle some of the major problems with conventional research methodologies. Scientists can create more efficient treatment plans that attempt to block or reduce the growth of AD by utilizing the special advantages of zebrafish. With the potential to benefit millions of people with AD and their families, this research holds great promise for better outcomes [3].

## Review

The causes and mechanisms of AD

**Figure 2:**
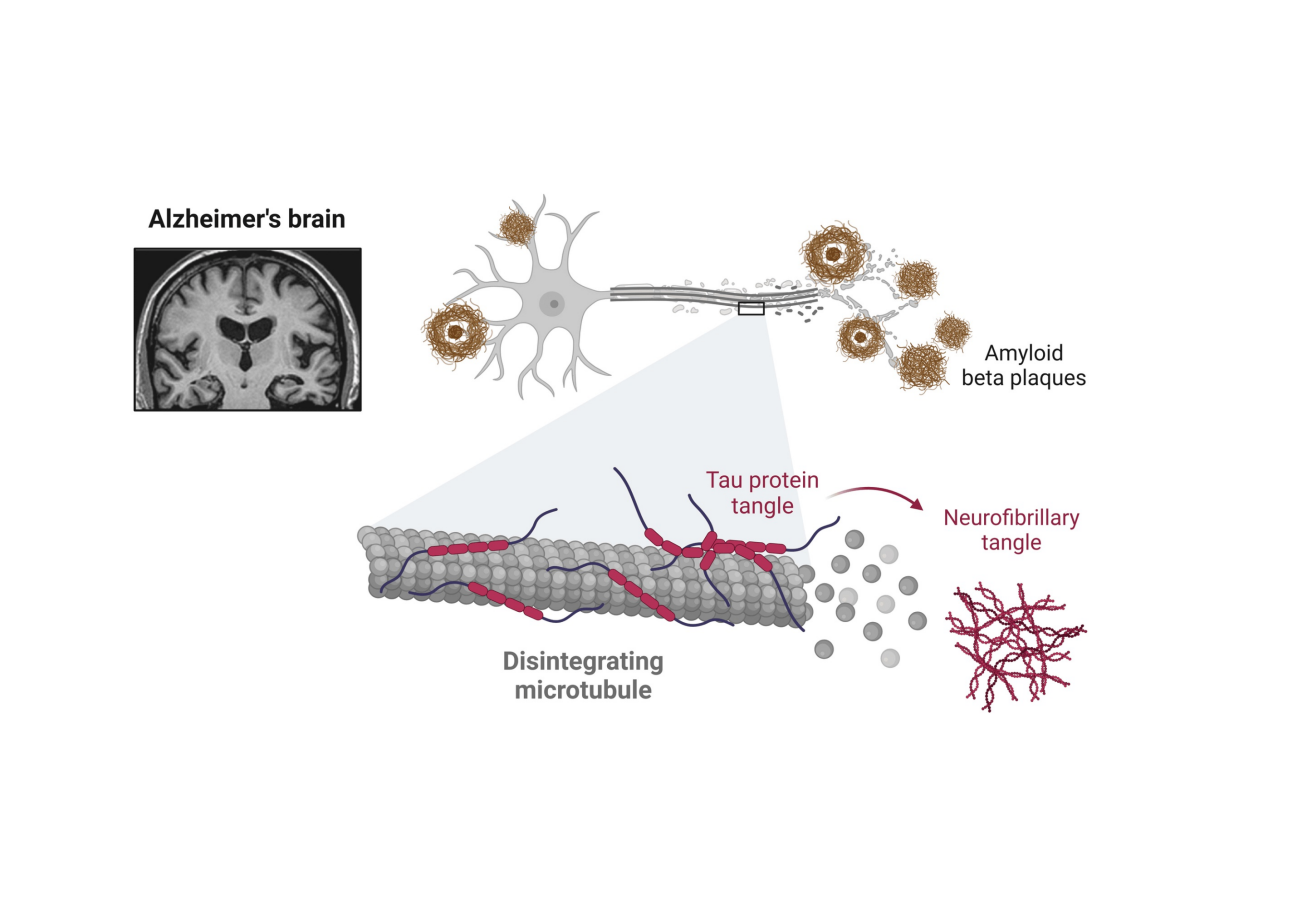
The prime pathophysiology of Alzheimer’s disease This image was originally produced by the author. Created with Biorender.com.

AD is typified by unique pathological characteristics pertaining to the hippocampus and cerebral cortex, particularly the frontal and temporal lobes. The two primary pathogenic features are the buildup of NFTs and Aβ plaques (Figure 2). These anomalies cause neurons to gradually deteriorate, synaptic dysfunction to occur, and ultimately, significant brain tissue loss.

The transmembrane protein known as amyloid precursor protein (APP) is widely expressed in the brain, is the source of the nic route of APP, and is cleaved by alpha-secretase, which halts the synthesis of Aβ peptides. In the amyloidogenic process, APP is initially cleaved by beta-secretase (BACE1), producing soluble and membrane-bound fragments that are further cleaved by gamma-secretase. This leads to the production of Aβ peptides, primarily Aβ40 and Aβ42. The most prevalent form of Aβ42 seen in amyloid plaques is more prone to aggregation. Aβ peptides group together to generate neurotoxic oligomers, which then further combine to create insoluble fibrils that build up outside of cells as amyloid plaques. These plaques cause inflammatory reactions and interfere with cell-to-cell transmission, which exacerbates neuronal damage and death [4].

In neurons, microtubules are crucial for intracellular transport and for preserving the cell’s structural integrity. Tau is a microtubule-associated protein that helps to stabilize these structures. When tau is hyperphosphorylated in AD, it loses its capacity to attach to microtubules and aggregates into paired helical filaments. Within the neuron, these paired helical filaments combine to form NFTs, which are mostly seen in the cell bodies and dendrites of afflicted neurons. The buildup of NFTs causes neuronal malfunction and cell death by compromising the transport mechanism inside the neuron.

Also linked to AD is persistent neuroinflammation. The presence of amyloid plaques activates microglia and astrocytes, the brain’s innate immune cells. These activated glial cells emit pro-inflammatory cytokines and other mediators that exacerbate neuronal damage. One of the first and most important events in AD is synaptic dysfunction, which is brought on by the buildup of NFTs and amyloid plaques. There is a substantial correlation between synaptic loss and the cognitive deterioration seen in AD patients. Significant neuronal loss occurs over time, especially in areas like the cerebral cortex and the hippocampus, which are essential for memory and cognition. Significant brain shrinkage is caused by extensive neuronal loss as the illness worsens. The cerebral cortex and the hippocampus show the most shrinkage, and the clinical symptoms of AD, such as memory loss, cognitive decline, and behavioral abnormalities, are linked to this decrease in brain volume.

Mutations in genes, including APP, PSEN1, and PSEN2, which raise Aβ synthesis or aggregation, are associated with FAD. Genetic risk factors impact SAD; the most important one is the apolipoprotein E (APOE) ε4 allele, which impacts the metabolism and clearance of Aβ. The complex pathophysiology of AD involves several interrelated processes, including the accumulation of Aβ plaques, the formation of NFTs, chronic neuroinflammation, synaptic dysfunction, and considerable neuronal death. The gradual cognitive decline and memory impairment that are hallmarks of AD are caused by these pathological alterations. Comprehending these pathways is essential for creating tailored treatments meant to reduce or stop the advancement of this crippling illness. 

The roles of autophagy and mitochondrial dysfunction in the pathogenesis of AD have come under more scrutiny in recent times. Neuronal health depends on autophagy, a cellular mechanism that breaks down and recycles damaged proteins and organelles. Because autophagy is frequently compromised in AD, misfolded proteins like tau and Aβ accumulate. This accumulation exacerbates the illness by causing neuronal damage and loss. The elimination of these harmful proteins is hampered by defective autophagy, which exacerbates the degenerative aspects of AD. There is much evidence to support the theory that autophagy failure is a major pathophysiological factor in AD. Research has demonstrated that people with AD have a considerable buildup of autophagosomes in their frontoparietal cortex, accompanied by a decrease in the autophagy-regulating protein beclin1. Genetic variants related to the lysosomal peptidase cathepsin D, which is involved in the clearance of tau protein and Aβ peptides, have been linked to an increased risk of AD and provide more evidence of the connection between autophagy and AD. Furthermore, the altered phosphatidylinositol-binding clathrin assembly protein, another AD susceptibility gene, negatively affects autophagy. In AD, autophagic vacuoles accumulate, and autophagosome formation and maturation are impaired due to lysosomal proteolytic failure, which is the primary cause of autophagy dysfunction. This leads to the buildup of tau and Aβ proteins, which are important indicators of AD. Studies reveal that autophagosomes include APP and active gamma-secretase complex components that can produce Aβ. Autophagy regulates both the formation and clearance of Aβ. Moreover, autophagy is necessary for the release of Aβ peptides into the extracellular space and the subsequent development of plaque. Studies have shown that reactivating autophagy can substantially increase Aβ secretion, which is considerably decreased when autophagy is compromised. Further, activating autophagy in AD mice with mTOR inhibitors such as rapamycin increases Aβ clearance and memory. When autophagy is impaired, hyperphosphorylated tau and insoluble tau species accumulate, which is why autophagy is essential for the clearance of tau. In AD, autophagy may be a therapeutic target for both the tau and Aβ pathologies since restoration of autophagy decreases tau phosphorylation and inhibits the production of NFTs.

Yet another important component of AD is mitochondrial dysfunction. The energy factories of cells, mitochondria, produce the energy required for neural activity. Through the process of oxidative phosphorylation, mitochondria produce adenosine triphosphate (ATP), which is necessary for the creation of energy in cells. The electron transport chain (ETC), a chain of protein-metal complexes (I-IV) in the mitochondrial membrane, is where nicotinamide adenine dinucleotide (NADH) enters to initiate this process. These complexes facilitate the transport of electrons, which activates ATP synthase (complex V) to stimulate the production of ATP.

It is becoming more widely acknowledged that altered mitochondrial functions, especially those connected to the ETC, occur in AD. Since these disturbances could be important in the development of AD, mitochondria are a prospective target for therapy in the early stages of the disease and a major area of current study. Reduced energy generation and increased oxidative stress are the results of damaged and inefficient mitochondria, which are common in AD. Stress has the potential to exacerbate cellular damage and accelerate neuronal death. Moreover, reactive oxygen species generation and an imbalance in calcium homeostasis are two more consequences of mitochondrial malfunction that are detrimental to neurons.

Cellular damage is produced by a vicious loop of defective autophagy and mitochondrial dysfunction interacting. For example, oxidative stress is caused by damaged mitochondria that are not adequately cleaned by autophagy, further impairing mitochondrial function. Developing innovative treatment techniques requires addressing these biological mechanisms. Through the promotion of autophagic activity or the restoration of mitochondrial function, scientists aim to lessen the cellular damage associated with AD and maybe even stop the disease’s progression. Gaining insight into and focusing on these pathways is a possible approach to creating AD therapies that work better [5].

An outline of the zebrafish model organism 

The zebrafish (*Danio rerio*), a small freshwater fish native to South Asia, is a species found in tropical regions. Due to their prolific year-round breeding, transparent outward development, and simplicity of care, they are a widely used vertebrate model in many scientific domains. The family *Cyprinidae*, which also contains barbs, carps, and true minnows, is home to zebrafish. The *Danio* genus (formerly Brachydanio, as morphological and genetic evidence suggested no need to separate the two genera) includes more than 40 related species, including zebrafish [6].

Zebrafish are usually kept in lab conditions at a temperature of about 28°C. However, wild strains have been reported to withstand temperatures ranging from 24.6°C to 38.6°C without exhibiting symptoms of heat stress, suggesting that they have a broad range of thermal tolerance. Zebrafish eat mostly insects, nematodes, and other zooplankton as part of their normal diet. Larger fish like snakeheads, needlefish, catfish, and knifefish are probably the main predators of zebrafish living in their natural habitats.

In biological research, especially in developmental biology and the study of human disorders, the zebrafish (*D. rerio*) has been shown to be an invaluable model organism. Streisinger first used zebrafish in a study in the 1970s, and there are a number of important reasons why they are a good choice for experiments [7].

Researchers may comfortably see and work with zebrafish embryos even at the one-cell stage because they are readily accessible. Numerous developmental and genetic studies can be made easier by this characteristic.

Optical Clarity

Because the zebrafish is developing, researchers can see in vivo developmental processes with remarkable accuracy and detail because of its optical clarity.

Diploid Genome

Zebrafish are particularly well-suited for genetic research and modification because of their diploid DNA.

According to large-scale forward genetic screenings carried out by scientists like Nobel Prize winner Christiane Nüsslein-Volhard and others, these qualities have made zebrafish an excellent model organism for developmental biology [7]. Beyond the field of developmental biology, the zebrafish model has been increasingly popular in the investigation of human illnesses. Since zebrafish reach sexual maturity at the age of 12 weeks and may produce hundreds of embryos per week, their rapid growth is very useful in this aspect. Zebrafish embryos are notable for their ability to grow in utero, which makes it possible to manipulate and observe them from the very beginning of their development through experimentation [8].

The striking similarities between the zebrafish (*D. rerio*) and the mammalian brain in terms of anatomy, function, and important neurotransmitter systems have made the zebrafish a useful model organism for research into neurodegenerative illnesses. Because zebrafish embryos contain a central nervous system (CNS) and completely formed main organs by 72 hours post-fertilization (hpf), they are especially valuable for early-stage studies on neurological diseases. Mammals and zebrafish have very similar brain architecture and neural circuitry, including preserved key neurotransmitter systems, including glutamatergic, GABAergic, histaminergic, cholinergic, and dopaminergic pathways. Researchers can see the CNS and other organs develop and operate in real time because of the developmental transparency of zebrafish embryos. Zebrafish may also be genetically modified using techniques such as CRISPR/Cas9, which enable the study of gene function and the development of disease models. It is now easier to investigate the precise genetic contributions to neurodegenerative illnesses because of the availability of transgenic lines and gene knockdown techniques (such as morpholinos). Zebrafish are less expensive to keep than mammalian models, and their high fertility and quick growth make it possible to test possible medicinal chemicals on a wide scale.

AD is used to study the formation of amyloid plaques and tauopathy; ALS is used to study the degeneration of motor neurons with introduced mutations in genes like SOD1 and TDP-43; and Huntington’s disease (HD) exhibits neurodegenerative phenotypes with mutant huntingtin (HTT) gene expression. As a result, using zebrafish as a model organism in ND research provides a strong foundation for identifying the underlying causes of these conditions and screening potential therapies. Zebrafish are an invaluable tool in the battle against neurodegenerative illnesses because they share many neurological and neurochemical systems with mammals and may be genetically modified. Zebrafish are also advantageous since they are transparent and inexpensive.

The zebrafish (*D. rerio*) is proving to be a useful model for translational research on neurological issues in humans. In this paper, we evaluate the significant neurological and behavioral similarities between humans and zebrafish. As a potent vertebrate model for examining neurodegenerative illnesses in humans, it has received extensive validation. There is a strong similarity between the neuroanatomical and neurochemical pathways in the brains of zebrafish and humans. There are documented commonalities between them in terms of their physiology, emotions, and social behavior patterns. Interestingly, tauopathy and the pathogenesis of AD have been successfully reproduced in zebrafish models [9].

Rationale for using zebrafish in AD research

In 2013, it was shown that the zebrafish and human genomes had a substantial amount of similarity and that 70% of human genes have zebrafish orthologs. This was made feasible by the extensive gene collection of the zebrafish genome sequencing effort. Based on the increasing usage of zebrafish illness models throughout time, which have data gathered from the literature available in the PubMed and Scopus databases (2000-2021), one may infer the rise of their application in translational research. Zebrafish models are highly intriguing because of their capacity to reproduce quickly, which enables researchers to produce stable disease model lines in as little as three to four months. Research on zebrafish can begin as soon as three days following fertilization because they are extremely prolific, laying 200 eggs or more in a clutch every two to three days [10].

It is far simpler to keep them in a lab setting than it is to replicate the circumstances necessary for mammals because their native environment is so simple. Therefore, it is possible to grow zebrafish economically. Because the larvae are tiny, it is easy to do high-throughput screening of neuroactive chemicals [11]. The rapid advancement of experiments is accelerated by their brief generation spans of three to five months. In a typical cellular context, it is quite simple to introduce transitory modifications of gene activity and then examine the results. mRNAs, transgenes, morpholino antisense oligonucleotides, and genome editing methods like CRISPR/Cas9 and TALENs 11, 12, and 13 are all highly flexible in manipulating embryos genetically. A number of the zebrafish’s genes are orthologs of those altered in human FAD, and they have a vertebrate neural structural organization. Ever since the zebrafish genome was fully sequenced, researchers have improved the online application for using gene ontology to analyze zebrafish genes in interesting research. 

Despite intensive research efforts, the disease known as AD is still a sickness that is poorly understood and difficult to treat. It is currently unclear whether precise processes account for most AD cases. Less than 1%, on the other hand, are linked to FAD, a type of illness that has an autosomal dominant inheritance pattern and an early start. On the other hand, nearly 99% of AD patients are categorized as having SAD. Genetic and environmental factors interact intricately to cause SAD. Allele ε4 of the apolipoprotein E gene (APOEε4) is the most important known contributor among the genetic risk factors. In addition to genetic predisposition, environmental variables are important in the onset and course of SAD, resulting in a complex and varied illness.

Comparative analysis with other models of AD

**Figure 3:**
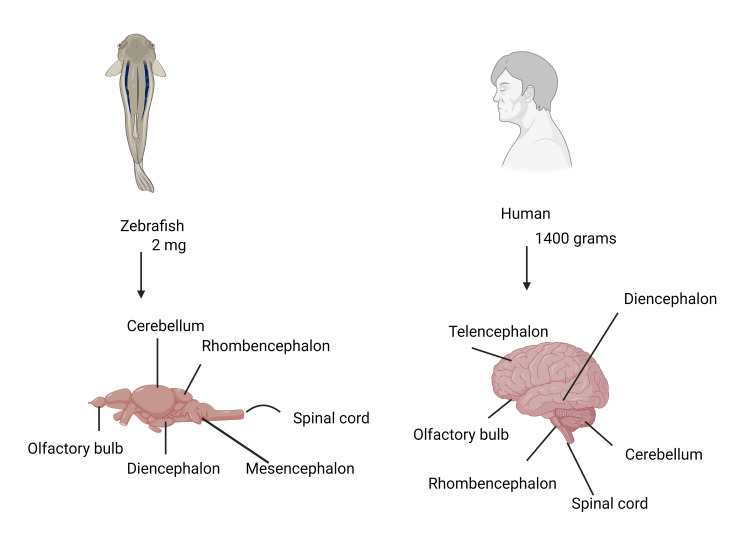
Comparison of a mid-sagittal section of the zebrafish brain with a mid-sagittal section of the human brain Comparison of brain weights between zebrafish and humans; the weight is expressed in milligrams (mg) for the zebrafish brain and in grams (g) for the human brain. This image was originally produced by the author. Created with Biorender.com.

Zebrafish (*D. rerio*) have become an important model organism for neurological and behavioral research because of a number of important characteristics that make them similar to humans. Zebrafish are useful because of their close phylogenetic relationship to humans. They have a large amount of the same genome as humans, which makes it possible to study behavior, genetics, brain function, and neurological disorders in a setting that is extremely relevant to human biology. The application of research findings from zebrafish studies to human health and illness is made easier by this evolutionary relationship. Furthermore, the olfactory bulb and hypothalamus, two fundamental organizational components of the human brain, are still present in the zebrafish brain. Notably, memory and spatial navigation are significantly aided by the lateral pallium in zebrafish, which is comparable to the hippocampus in mammals. Because of these preserved anatomical features, scientists may study how certain brain areas develop and function in zebrafish, using this model to better understand brain problems in humans. As a result, zebrafish provide an invaluable platform for investigating the processes behind behavioral and neurological abnormalities and trying out possible therapeutic approaches (Figure 3).

Zebrafish and animals contain similar neurotransmitter systems, including glutamatergic and GABAergic pathways. Furthermore, their neurotransmitter transmission processes are similar to those of mammals, as evidenced by the presence of muscarinic cholinergic receptors in their brain extracts. Apart from these commonalities, the CNS of zebrafish includes several cell types that are also present in the brains of mammals, such as oligodendrocytes, motor neurons, myelin, astrocytes, microglia, and cerebellar Purkinje cells. The cellular connections and functions that are important to human neurobiology may be thoroughly studied thanks to these molecular analogies. The blood-brain barrier (BBB), which is composed of tight junctions that control the permeability of macromolecules, is another essential characteristic of zebrafish. In order to learn more about neuroprotective compounds and drug delivery methods and perhaps develop therapies for humans, it is essential to have regulated permeability. Zebrafish are an excellent model for furthering neuroscience research and creating treatments for neurological illnesses because of their special mix of evolutionary closeness, preserved brain structure, neurotransmitter systems, cellular makeup, and BBB features. Zebrafish research can, therefore, provide important new insights into the basic mechanisms behind brain health and illness as well as highly applicable discoveries to human circumstances. By taking advantage of these benefits, researchers can accelerate the creation of efficient therapies for neurological conditions and expand our knowledge of the nervous system [12].

Advantages of the zebrafish model for AD induction

The zebrafish, or *D. rerio*, model is a helpful model for AD research because they have some benefits over mouse models, even though mice lack sophisticated cognitive functions. Zebrafish are very receptive to targeted gene knockouts and the production of transgenic lines, which makes them one of the most advantageous fish genetically. This feature is especially helpful when researching the effects of inherited risk factors like APOE4 and certain genes linked to AD, such as PSEN1, PSEN2, and APP.

Zebrafish may produce a huge number of progeny due to their rapid growth cycle and high rate of reproduction. Large-scale genetic screens are made possible by this, which also makes the research of gene-environment interactions and the generational implications of genetic changes easier. Zebrafish embryos also have the benefit of being transparent, which makes it possible to use a variety of imaging tools to view pathological alterations and brain development in real time. This characteristic is particularly useful for examining the early development and course of disease associated with AD.

It is significant to note that tau protein tangles and Aβ plaques, two pathogenic features of AD that are also seen in human AD, are retained in zebrafish. Because of their similarities, they may be used to assess possible treatment approaches and investigate the molecular processes underlying these characteristics. Zebrafish exhibit a variety of observable activities, including learning and memory tests, locomotor activity, and social interactions, that can be impacted by AD disease while having simpler cognitive capacities. These behavioral tests offer insightful readouts for evaluating the results of genetic and pharmacological therapies.

Zebrafish are also the perfect choice for high-throughput drug screening due to their tiny size, high fertility, and adaptation to microplate formats. The discovery of substances that could affect the pathophysiology or symptoms of AD is sped up by this capacity. All things considered, these qualities make zebrafish a useful model for expanding our knowledge of AD and creating novel treatment strategies [13].

Novel techniques for zebrafish AD modeling

A Revolutionary Approach to Healthcare

Customizing treatment to meet the specific requirements of each patient is the main goal of personalized medicine. This approach has great potential, particularly for people with ailments for which there are no suitable medicines at this time. Both prevalent illnesses with heterogeneous characteristics and uncommon, unidentified conditions require personalized therapies. These tactics seek to identify the fundamental causes of illnesses and create focused treatments. Zebrafish have become appealing subjects for neurological research because of their transparent embryos, which allow for real-time monitoring of CNS development, as well as their brain architecture and synaptic networks that are similar to those of higher vertebrates. They also demonstrate complex behaviors quickly after fertilization. Their preclinical pharmacological research usefulness is further enhanced by their tiny size, excellent fertility, and adaptability for high-throughput drug screening. Because of these traits, as well as their genetic similarity to humans and consistent phenotypic outcomes, zebrafish serve as a dependable model for translational research, helping to close the gap between basic science and real-world applications.

The goal of personalized medicine is to tailor care to the particular genetic, environmental, and behavioral characteristics of each patient. For illnesses like AD, where conventional therapies frequently prove ineffective, this strategy is very beneficial. In order to create customized treatment solutions for neurological illnesses, zebrafish are now an essential model organism. With the use of TALENs and CRISPR/Cas9 technologies, zebrafish embryos may be genetically engineered to produce accurate models of human genetic diseases, such as FAD. Zebrafish engineering, for instance, may be used to produce human AD-associated genes, including APP, PSEN1, and PSEN2, which can provide insights into the illness.

Zebrafish embryos are transparent, making it possible to view the development of cells and tissues in real time, including the brain. This allows researchers to examine AD pathophysiology from the very beginning. In living zebrafish, researchers can see how amyloid plaques and NFTs form, offering dynamic insights into disease mechanisms. Zebrafish are useful for researching the effects of AD and possible therapies because they display a variety of activities that can be objectively examined, including learning, memory, social interactions, and motility. Behavioral tests, such as the new object recognition and T-maze tests, assess cognitive function in zebrafish models of AD.

Zebrafish are very fertile and compact, which makes them ideal for high-throughput drug screening. It is possible to test hundreds of embryos at once, allowing for the quick assessment of several chemicals. Drugs that prevent, lessen, or ameliorate the harmful consequences of NFTs and amyloid plaques can be found using zebrafish screening. With the use of CRISPR/Cas9 technology, the zebrafish genome can be precisely edited to add or fix mutations linked to AD, resulting in models that closely resemble the genetic makeup of AD in humans. To investigate the function of human APOE-4 ε4 alleles in AD risk and progression, CRISPR can be used to introduce these alleles into the genome of zebrafish.

To explore the accumulation and consequences of human AD-related proteins in the brain, researchers can create transgenic zebrafish lines that produce mutant variants of tau or APP. Real-time observation of plaque development and removal is made possible by transgenic zebrafish that express GFP-tagged Aβ. To investigate the functional ramifications of AD-implicated genes, morpholinos, artificial chemicals that inhibit certain gene expression in zebrafish, can be used to knock them down. Researchers can analyze AD pathogenesis pathways by silencing genes related to tau phosphorylation or amyloid processing.

Controlling neuronal activity using chemical or light-activated proteins is the goal of optogenetics and chemogenetics. To investigate particular neural circuits impacted by AD and evaluate possible treatment approaches, these methods may be used on zebrafish. Researchers can investigate the correlation between neuronal activity and symptoms of AD by manipulating neurons in the brains of zebrafish.

Researching AD in an innovative way presents a number of hurdles, including reproducibility, precision, and accuracy problems. There may be challenges in analyzing complicated data and integrating these methods with current technologies. The issue is made worse by ethical and legal obstacles, including acquiring clearances, data protection, and patient permission. Budgetary restrictions and the requirement for specialized training might make adoption and accessibility more difficult. The variability of AD further means that innovative methods must take individual characteristics and a range of clinical presentations into consideration. The therapeutic effects and long-term efficacy remain uncertain. Overcoming these challenges will require teamwork and meticulous validation in order to guarantee that these advancements expand our knowledge of AD and how to treat it [14].

Current challenges and future perspectives

There are significant disadvantages to take into account while employing zebrafish (*D. rerio*) in neurological research, despite their many benefits. The intricacy of human illnesses, which can entail complicated physiological processes and a range of symptoms that may not be fully replicated in zebrafish models, is one major obstacle. Because of the disparity in physiology and illness presentation between humans and zebrafish, it may be more difficult to directly apply the findings of zebrafish studies to human clinical situations.

Utilizing zebrafish models to investigate AD is one illustration of its potential. The processes of Aβ buildup and its consequences on neuronal health were studied in a noteworthy work using transgenic zebrafish expressing human Aβ. This model enabled high-throughput screening of possible therapeutic chemicals and provided real-time observation of Aβ plaques and neuronal damage, offering insights into the course of the illness. Zebrafish have been shown to be useful in neurological research, as evidenced by the study’s ability to efficiently simulate some features of AD and promote treatment discovery.

As a model organism for researching AD, zebrafish have several benefits, but they also have significant drawbacks. The simplicity of pharmacological manipulation by chemical addition to the water is one of its main benefits. However, due to varying absorption through the gills and skin, it might be difficult to pinpoint the exact amount of chemicals entering the fish. Furthermore, nothing is known about the zebrafish-specific Aβ peptide, and further investigation is required to ascertain whether or not zebrafish post-translationally process APP similarly to humans. Zebrafish are remarkable because, in contrast to humans, they have the capacity to repair neurons along the rostrocaudal brain axis throughout their lives. Research has demonstrated that zebrafish microinjected with Aβ1-42 peptide display neurogenesis, encompassing the growth of neural cells. Studies comparing juvenile and adult fish have shown that microglia are activated to counteract Aβ-induced neurodegeneration by promoting neurogenesis and preventing synaptic degradation. This implies a possible connection between neurogenesis, neuroinflammation, and neurodegeneration. Zebrafish as an AD model may face challenges due to their regenerative capacity, but this also provides fresh opportunities to study the molecular signaling pathways involved in neuron regeneration. Our knowledge of the molecular processes necessary for the regeneration of the mammalian CNS may be improved by this research.

Restrictions pertaining to technology constitute another limitation. Zebrafish provide a strong platform for investigating genetic and neurological processes, but to fully realize their potential, more progress in data processing, genetic tools, and imaging technologies is required. The development of zebrafish imaging technology is another crucial path. More precise monitoring of neuronal growth, disease progression, and treatment responses at the cellular and molecular levels will be possible with the use of high-resolution, real-time imaging tools. Computational advancements, along with imaging innovations, will enable more thorough data processing and interpretation. It will also be essential to create more advanced behavioral testing. Through developing and improving assessments to gauge cognitive abilities, social interactions, and other intricate behaviors, scientists may enhance their comprehension of the impact of neurological disorders on zebrafish and, more accurately, assess the effectiveness of possible therapies. In order to better simulate neurological illnesses in humans, future research should concentrate on creating more advanced genetic and pharmacological techniques, improving zebrafish models, and encouraging cooperative efforts to convert these discoveries into therapeutic approaches. Nevertheless, zebrafish remain a powerful model for creating customized neurology treatments. Their ability to control genes, see embryonic development in a transparent way, have similar brain regions, and can be used for large-scale drug screening makes them essential for understanding the etiology of disease and developing personalized treatments. As research progresses, zebrafish will play a critical role in bridging the gap between lab data and clinical applications, ultimately improving the precision and effectiveness of neurological medications.

Collaboration between researchers, doctors, and industry partners will also be crucial. These sorts of collaborations might speed up the translation of zebrafish research findings into clinical applications, such as the development of novel therapies and specialized treatment regimens. This collaborative approach will ensure that human health benefits from zebrafish model research and can lead to effective, practical medicines.

Lastly, exploring the potential use of zebrafish models to study rare and complex brain diseases may open up new avenues for study and therapeutic advancement. By expanding the scope of zebrafish research to include a wider spectrum of neurological issues, scientists might uncover new insights and develop customized remedies for a greater variety of conditions [15].

Zebrafish as a tool for neurological disease prediction: adaptability, advancement, and enhanced diagnosis

Preclinical research for medication repurposing, drug discovery, and the early stages of drug research are all encouraged by understanding molecular mechanisms of pathology and examining genetic variations associated with neurological disorders using zebrafish and zebrafish technology.

The zebrafish, namely the *D. rerio* species, are increasingly being used as models for NDs and to find novel therapeutic agents. Zebrafish and humans have around 70% of the same genome, and 84% of the genes associated with human disease have zebrafish counterparts. Zebrafish are a good choice for vertebrate models because of their short generation periods, ease of care, and affordability. Through the use of external fertilization, they generate a huge number of embryos and larvae that are optically transparent and perfect for non-invasive imaging and high-throughput analysis. According to Saleem and Kannan (2018), zebrafish models of AD have neurological, behavioral, and pathological characteristics similar to those of human AD [16]. However, measuring substances taken in through the skin and gills is difficult, and studying neurodegeneration is made more difficult by these substances’ capacity to repair neurons [17].

Despite these challenges, tau-expressing transgenic zebrafish show both tau-dependent neuronal death and significant pathogenic features of tauopathies. Upon injection into zebrafish embryos, the amino acid 42 induces symptoms akin to those of AD, including tau phosphorylation increases, memory loss, and cognitive abnormalities. According to Nada et al. (2016), okadaic acid causes deficiencies in learning and memory by elevating tau phosphorylation and Aβ-plaques [18].

Behavioral assays for assessing cognitive function in zebrafish

Executive function impairments include abnormalities in brain flexibility and memory retention, which are connected to a wide spectrum of neurological and mental illnesses. The development of animal models with trustworthy behavioral evaluations would be necessary to target these cognitive deficiencies and perhaps aid in the development of effective drugs for a variety of disorders.

Executive function tests are few despite the fact that zebrafish models hold great potential for studying complex brain disorders. A series of consecutive two-choice discriminations is used to provide analysis for the free-movement pattern (FMP) Y-maze, which incorporates aspects of the traditional Y-maze experiment. This exam’s validity as a gauge of memory storage and memory retention has been confirmed by a comparison of task performance metrics in mature zebrafish given various medications known to impede these mental abilities. The striking parallels in maze navigation across *Drosophila*, mice, and humans utilizing modified versions of the test proved the task’s cross-species validity. The FMP Y-maze is a delicate tool with remarkable translational usefulness for evaluating memory storage and mental flexibility across species. Intellectual and motor functioning, as well as attention and responsiveness, are often negatively impacted by sleep deprivation (SD). There is strong evidence that SD causes disruptions to the neuro-immuno-endocrine system, which is linked to cognition.

The organizational and functional characteristics that zebrafish share with other vertebrates make them highly valuable for rapid screening. A study was conducted on sleep-deprived zebrafish to observe the effects of aspirin, also known as acetylsalicylic acid, on their motor and cognitive abilities. Partition preference and swimming time in spinning exercises were employed to evaluate locomotor activity, while a T-maze was utilized to assess learning and memory. Zebrafish models have been shown in behavioral pharmacology research to be effective in quickly screening chemicals for potential medications as well as in identifying important pathways that might result in the development of innovative treatment methods for neurobehavioral illnesses [19].

CRISPR/Cas9-mediated zebrafish gene modification

**Figure 4:**
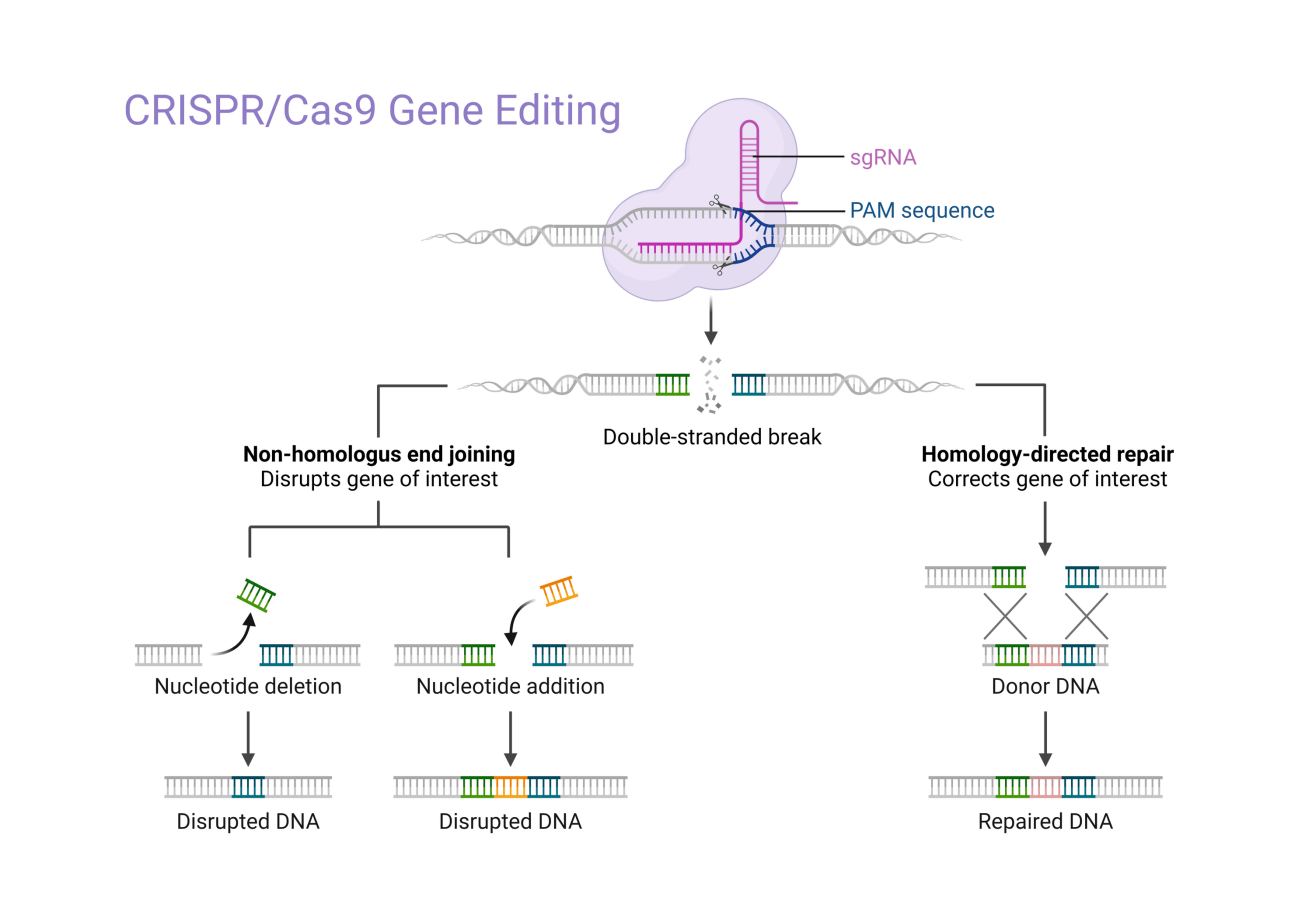
CRISPR/Cas9-mediated gene modification This image was originally produced by the author. Created with Biorender.com.

The CRISPR/Cas9 genome editing technology has rapidly evolved over the past few years, with a significant increase in knock-out reports, especially in the zebrafish community. However, insertion studies have been more challenging. CRISPR/Cas9, which is taken from the immune system and adapts to prokaryotic species like bacteria and fungus, is made up of three important components: trans-activating crRNA (tracrRNA), endonuclease Cas9, and CRISPR messenger RNA (crRNA). The domains that comprise Cas9, which cleaves target DNA, include REC I, REC II, bridge helix, protospacer adjacent motif (PAM)-interacting domain, HNH, and RuvC. The crRNA-tracrRNA duplex can be fused to produce a single guide RNA (sgRNA), which has a guide sequence that is 20 nucleic acids shorter than the target area. With the help of the sgRNA, Cas9 creates a double-stranded break (DSB) at the target location (Figure 4).

Over the past few years, the CRISPR/Cas9 genome editing technology has advanced quickly, and there have been a lot more reports of knockouts, particularly in the zebrafish community. Insertion studies, however, have proven more difficult. CRISPR/Cas9, an adaptive immune system found in prokaryotes (bacteria and archaea), is composed of three fundamental components: the endonuclease Cas9, crRNA, and tracrRNA. PAM-interacting domain, RuvC, bridge helix, REC I, REC II, and HNH make up the target DNA-cleaving enzyme Cas9. The crRNA-tracrRNA duplex can be fused to produce a sgRNA, which includes a 20-nucleotide guide sequence complementary to the target region. With the help of the sgRNA, Cas9 creates a DSB at the target location [16]. 

Non-homologous end joining (NHEJ) and homology-directed repair (HDR) are the two primary methods used to treat these fractures. HDR is less effective but allows for more precise repair using a donor DNA template. NHEJ is more effective for gene disruption since it is error-prone and frequently causes insertions or deletions (indels). Target sequences can be cleaved with great efficiency when sgRNA and Cas9 are expressed together.

There are two age categories for AD: late-onset AD (LOAD) and EOAD. EOAD, which often appears before the age of 65, is frequently caused by dominantly inherited mutations in presenilin-1 (PSEN1), presenilin-2 (PSEN2), and APP. Worldwide, more than 400 mutations affecting the amounts of Aβ production and leading to elevated Aβ42 or altered Aβ42/40 ratios have been reported. LOAD, which often manifests beyond the age of 65, has a more intricate and poorly understood pathogenesis, with several genetic loci and environmental variables playing a role in the disease’s establishment. Genome-wide association studies have linked over 20 genetic loci, including apolipoprotein E (APOE), to an increased risk of LOAD. 

Increased Aβ42 generation has been shown in vitro by site-directed mutagenesis investigations, which is consistent with in vivo results. Aβ42/40 ratios are higher in notable APP gene variants such as V717I and KM670/671NL (“Swedish APP” for HEK293). Given that CRISPR/Cas9 can fix mutations in brain cell genomes, it presents a promising treatment approach for AD. Using gRNA synthesis, this technology has been utilized to build models of PSEN1 and APP mutations, demonstrating accurate genome editing techniques. CRISPR/Cas9 offers a new path for AD research since it can target different genes in various cells, models of animals, or lines of cells. The knowledge gathered from the research projects on the workings and prospective uses of CRISPR/Cas9 in genome editing for in vivo and in vitro studies on AD may contribute to the creation of diagnostic and treatment instruments for NDs [20].

Optogenetic techniques for manipulating neural circuits in zebrafish

It is essential to understand how neural circuits cooperate to carry out calculations in order to comprehend how the brain produces actions. Since many cell types make up neural circuits, it is crucial to examine how these cells interact with one another. Genetically encoded calcium indicators (GECIs) and optogenetics, two recent developments, offer potent tools for precise timing and controlling neuronal activity. In optogenetics, genetically encoded chemicals are used to regulate the electrical activity of certain neurons by applying light pulses to those neurons when they are produced. These substances, referred to as microbial opsins, depend on the type of opsin that is employed to either activate or mute neurons [21].

In the area of optogenetics, channelrhodopsins, halorhodopsins, and archaerhodopsins are essential elements that allow for the exact regulation of neural activity using light. When exposed to blue light, channelrhodopsins, which include ChR2 and CatCh, are inward cation channels generated from green algae that depolarize neurons and cause action potentials. Halorhodopsins are inward chloride pumps from archaeal species that, when exposed to yellow light, hyperpolarize and quiet neurons. Examples of these pumps include Halo and NpHR. In addition to causing hyperpolarization in response to green or yellow light, archaerhodopsins, such as archaerhodopsin-3 (Arch) and ArchT, are light-driven outward proton pumps that effectively silence neurons in vivo. Through the precise manipulation of brain activity made possible by optogenetics, scientists may examine the roles played by individual neurons and neural networks, look into new therapies for neurological conditions, and examine the behaviors of animals. In addition to these optogenetic instruments, calcium imaging methods, such as synthetic calcium dyes and GECIs, provide information about neuronal activity by demonstrating alterations in intracellular calcium levels linked to synaptic activity and action potentials [22].

Research voids and novel concepts

Neuronal activity in several brain areas and animal models has been seen using GECIs. Long-term in situ neural activity imaging is made possible through them, which aids in the understanding of the many roles played by neurons and circuits in the regulation of behavior and brain function. More developments in GECI technology have improved its sensitivity, kinetics, and signal-to-noise ratio, which increases its usefulness for neuroscience studies. The research on brain circuits has been completely transformed by the discovery and use of optogenetics and GECIs. Researchers may analyze the intricate relationships among neural networks that underpin behavior and cognition by using these instruments, which provide accurate control and monitoring of neuronal activity. These innovations have enormous potential to improve our understanding of the brain and develop novel therapies for neurological diseases. Zebrafish models have lately become a viable study option for AD, providing special benefits that enhance conventional mammalian models. Zebrafish and humans have a striking resemblance in their genetic composition and architecture of the CNS, which is a considerable benefit. Because of these similarities, scientists are able to investigate complicated brain conditions like AD in a model system that closely resembles human biology.

Another important characteristic that helps with AD research is the transparency of zebrafish embryos in the early stages of development. This openness allows researchers to track the evolution of the illness in real time and provides information on how important pathological characteristics of AD-related conditions, including NFTs and amyloid plaques, arise. Understanding the underlying mechanisms of AD and testing potential treatment strategies are made possible by the non-invasive visualization of these processes. Zebrafish are ideal for high-throughput drug screening because they reproduce quickly and generate a huge number of progeny. The capacity to screen several compounds at once speeds up the drug development process. This could result in the identification of novel therapeutic options for the management of AD. Additionally, it is possible to genetically modify zebrafish to express human AD-related genes, allowing scientists to investigate how these genes affect the course of the illness. Researchers may study the molecular processes behind AD and assess possible targets for therapeutic intervention by introducing particular genetic mutations linked to the illness into zebrafish.

Zebrafish models provide advantages, but there are drawbacks to AD research as well. They don’t completely represent the intricacy of the illness as it manifests in humans, even if they faithfully mimic several features of AD pathology. To guarantee their applicability and dependability, results from zebrafish models must be validated using mammalian models and human clinical trials. Furthermore, because of their restricted behavioral repertoire, zebrafish are difficult to evaluate for cognitive function and memory deficiencies. Creating standardized behavioral tests customized for zebrafish models is essential to precisely describing symptoms associated with AD and assessing the effectiveness of possible therapies. Zebrafish-based AD research will need to advance through multidisciplinary collaboration between researchers, clinicians, and pharmaceutical businesses. Genetics, neurology, and drug discovery are just a few of the many scientific fields that may be integrated to answer complicated research concerns and hasten the development of successful therapies for AD. Furthermore, when zebrafish models are paired with cutting-edge technologies like CRISPR/Cas9 genome editing and advanced imaging techniques, they will be even more helpful in AD research. These technologies enable high-resolution imaging of disease processes and precise genetic alteration, providing new insights into the pathophysiology of AD and potential therapy targets. 

To translate zebrafish research findings into therapeutic applications, it will ultimately be necessary to create strong preclinical validation pipelines and biomarker identification platforms. Personalized treatment options for people affected by AD can be facilitated by combining human biomarker investigations with longitudinal studies in zebrafish models to uncover viable therapeutic targets. All in all, zebrafish models have fascinating prospects for expanding our knowledge of AD and creating successful treatments that enhance patient outcomes.

## Conclusions

Zebrafish can also be used to assess the effects of medication treatments and genetic alterations on memory and cognition because of their complex behaviors, which are statistically measurable. These behavioral investigations are essential to simulate the cognitive abnormalities associated with AD. Zebrafish are superior to mammalian models in both practical and ethical aspects. They are more accessible to a larger range of research centers because of their reduced space requirements, lower maintenance expenses, and lack of ethical problems. Researchers may examine the impact of genetic and environmental variables on brain development and neurodegeneration over a shorter timescale in zebrafish because of their fast development from embryo to adult, which provides insights into the early stages of AD pathogenesis.

Accurate genetic modification in zebrafish is made possible by methods like CRISPR/Cas9, which makes it easier to create models that closely resemble AD circumstances in humans. This aids in the understanding of the functions of certain genes in AD and the development of gene-targeted treatments. Zebrafish models also enable quick hypothesis testing and provide early high-throughput data, which is a supplement to established mammalian models. Research can be streamlined by validating positive results in zebrafish in more intricate mammalian systems.

For zebrafish research to reach its full potential in AD, multidisciplinary collaboration must be encouraged. By combining knowledge from the fields of computational biology, pharmacology, genetics, and neuroscience, we may develop a more thorough understanding and treatment plan for AD. For example, the discovery of novel therapeutic targets can be accelerated by combining sophisticated genetic editing tools with high-throughput drug screening approaches. Structured collaboration frameworks can help to alleviate the issues that come with working across disciplines, such as harmonizing approaches and communication. To make significant advancements in AD research, we fervently urge the scientific community to give priority to and finance these kinds of partnerships.

Conclusively, the zebrafish model is an effective instrument for studying AD, providing distinct benefits that enhance conventional mammalian models. Its genetic significance, capacity for high-throughput screening, and adaptability for in vivo imaging and behavioral investigations are its potential sources of influence. Zebrafish are a valuable tool for studying AD because of their capacity to shed light on early development, practicality, and ethical advantages. By making the most of these advantages, scientists may expand our knowledge of the pathophysiology of AD and hasten the creation of efficient therapies, both of which will benefit Alzheimer’s patients in the long run.
